# Supplementary material for: Integrin beta 1 facilitates non-enveloped hepatitis E virus cell entry through the recycling endosome
Source: Nat Commun. 2025 Jun 26;16:5403. doi: 10.1038/s41467-025-61071-y (PMC12202797; doi:10.1038/s41467-025-61071-y)
Supplement: Supplementary file 1 — Supplementary Information [file 41467_2025_61071_MOESM1_ESM.pdf]

Supplementary Material Fu et al.

Supplementary Methods

Primary human hepatocytes
Primary human hepatocytes (PHH) were purchased from PRIMACYST (Lot #: CHM2225-HE-Z) and seeded onto collagen-coated 24-well plate (PRIMACYST) following the manufacturer's protocol precisely. In brief, frozen vials of PHH were thawed in thawing media (PRIMACYST) and 4.1×10^5 cells per well were seeded in plating medium (PRIMACYST) and allowed to attach for 6 h at 37 ℃ and 5% CO2. After cells were attached, the media was removed and cells were washed twice with warm PBS and replenished with culture medium (PRIMACYST). 24 h post-seeding, the cells were treated with the indicated inhibitors and infected with nHEV at MOI = 0.5 GE/cell.

Renilla luciferase replication assay
S10-3 cells were electroporated with the RNA encoding a subgenomic HEV replicon[1] and seeded onto 12-well plates. Cell culture supernatant was harvested every 24 h and passed through a 0.45 µm Millex HV filter (Millipore), and subsequently stored at -80 °C. Fresh medium was then added to the culture, and the incubation process was resumed. Upon completing the experiment, all collected media were thawed at the same time, and 20 µl from each sample was subjected to a luciferase activity assay using the Renilla luciferase assay system (Promega). 50 µl of culture medium was added to individual wells of a black flat-bottom 96-well plate (Corning). Subsequently, 1 nM Coelenterazine was diluted in PBS and 60 µl per well was added. The emission was measured 10 s after the addition of the substrate with a Centro LB 963 Microplate luminometer (Berthold).

Structural visualisation of the DGR motif
Molecular structures were analysed and visualized using PyMOL (version 3.0, Schrödinger, LLC). The protein structures of HEV GT3 ORF2 (PDB ID: 2ZTN) was obtained from the Protein Data Bank. Structural features such as binding motif, secondary structures and interactions were examined and highlighted using PyMOL's built-in tools. For illustration purposes, cartoon representations were generated, and specific residues or regions were labelled accordingly. Any measurements, such as distances between atoms or angles, were also conducted using PyMOL's measurement functions.

Immunofluorescence and microscopy
S10-3 cells were seeded onto coverslips and fixed in 4% PFA and permeabilised in 0.1% Triton-X100 (Sigma) 24 h post-seeding. The cells were blocked in 1% bovine serum albumin (BSA) (Sigma) and immunostained with the following antibodies for 1 h at room temperature: mouse α-ITGB1 1:100 (Santa Cruz); rabbit α-ITGA2 1:200 (Abcam); rabbit α-ITGA3 1:200 (Millipore); rabbit α-ITGA5 1:200 (Abcam). The respective Alex-conjugated secondary antibodies were used at 1:1000 diluted in 1% BSA and incubated at room temperature for 1 h. Finally, the coverslips were mounted using the ProLong™ Glass Antifade Mountant (Thermo Fisher Scientific) and cured for at least 24 h in the dark. Confocal images were acquired using a Zeiss Airyscan LSM900 confocal microscope with 63x and/or 40x objectives, as indicated in the figure legend. For colocalisation studies, maximum projections of 3-4 z-slices were shown. Intensity plot profiles were generated using the ImageJ software. Colocalisation of

fluorescence images of single slices by confocal microscopy were analysed by evaluating Manders' coefficient using the "Colocalisation" module in Zen 2.1. 20 - 30 cells were analysed for each experimental condition and presented as violin plots.

Supplementary Figures

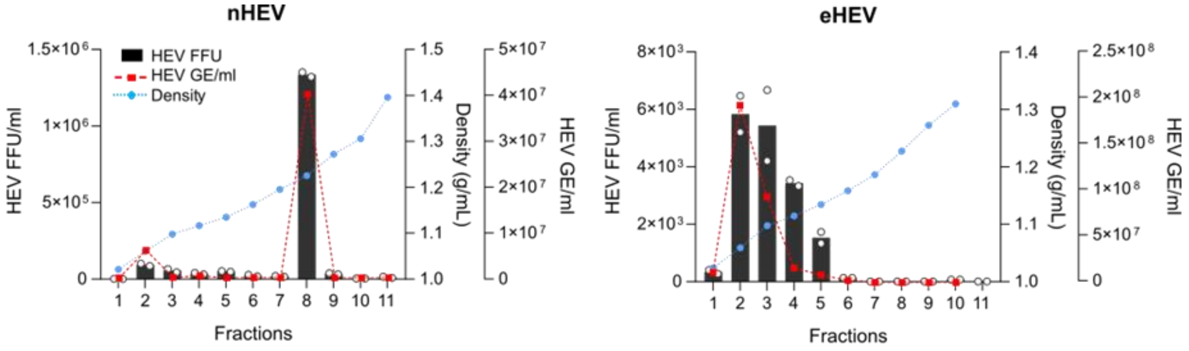

Supplemental Figure 1. Characterization of nHEV and eHEV. Buoyant density of HEV virions in cell lysates (left panel) or released into supernatant fluids (right panel) of S10-3 cells electroporated with HEV RNA purified through isopycnic iodixanol gradient centrifugation. HEV RNA in fractions was determined by qRT-PCR. HEV infectivity was determined by infecting S10-3 cells and quantifying FFU 5 days post-infection. Images of infected wells were taken with a Zeiss CellDiscoverer 7 microscope and the number of FFU was counted manually. n = 2 replicates from one virus production.

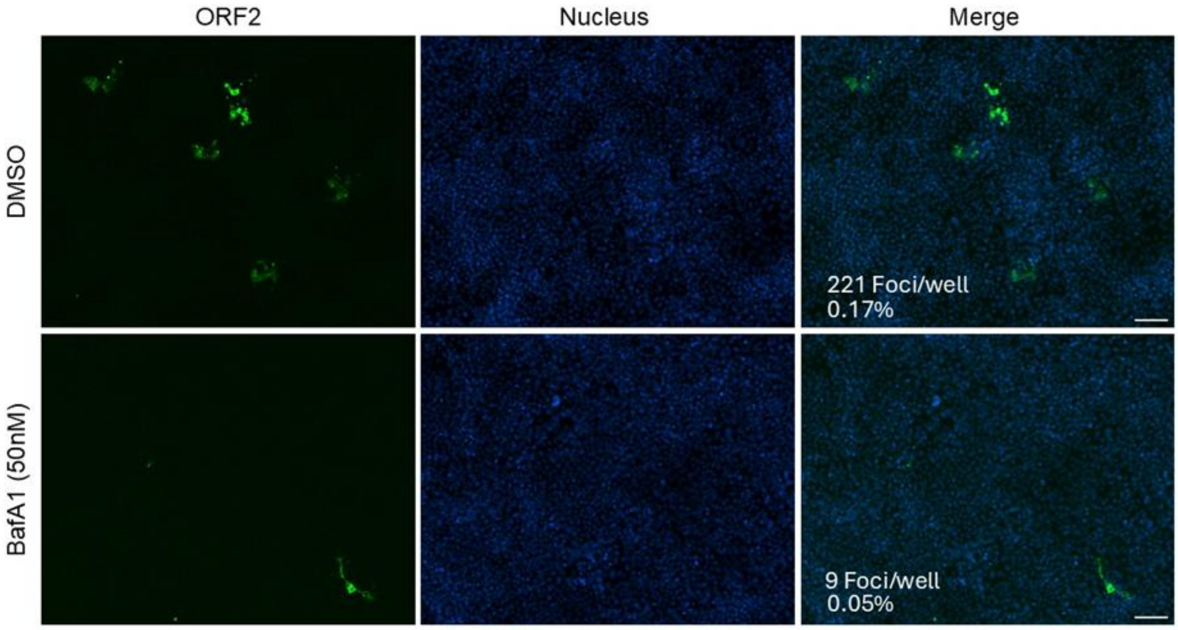

Supplementary Figure 2. Percentage of HEV infected cells upon BafA1 treatment. S10-3 cells were treated with 50 nM of bafilomyin A1 (BafA1) or DMSO for 30 min prior to infecting with nHEV (MOI = 0.1 GE/cell). Drugs and virus were removed after 24 hours and HEV infection was quantified by counting ORF2-positive FFUs as well as percentage of ORF2-positive cells 5 days post-infection. Images of infected wells were taken with a Zeiss CellDiscoverer 7 microscope. The number of FFU was counted manually and the percentage infection was quantified using ImageJ (version 1.54k). Scale bar = 100 µm.

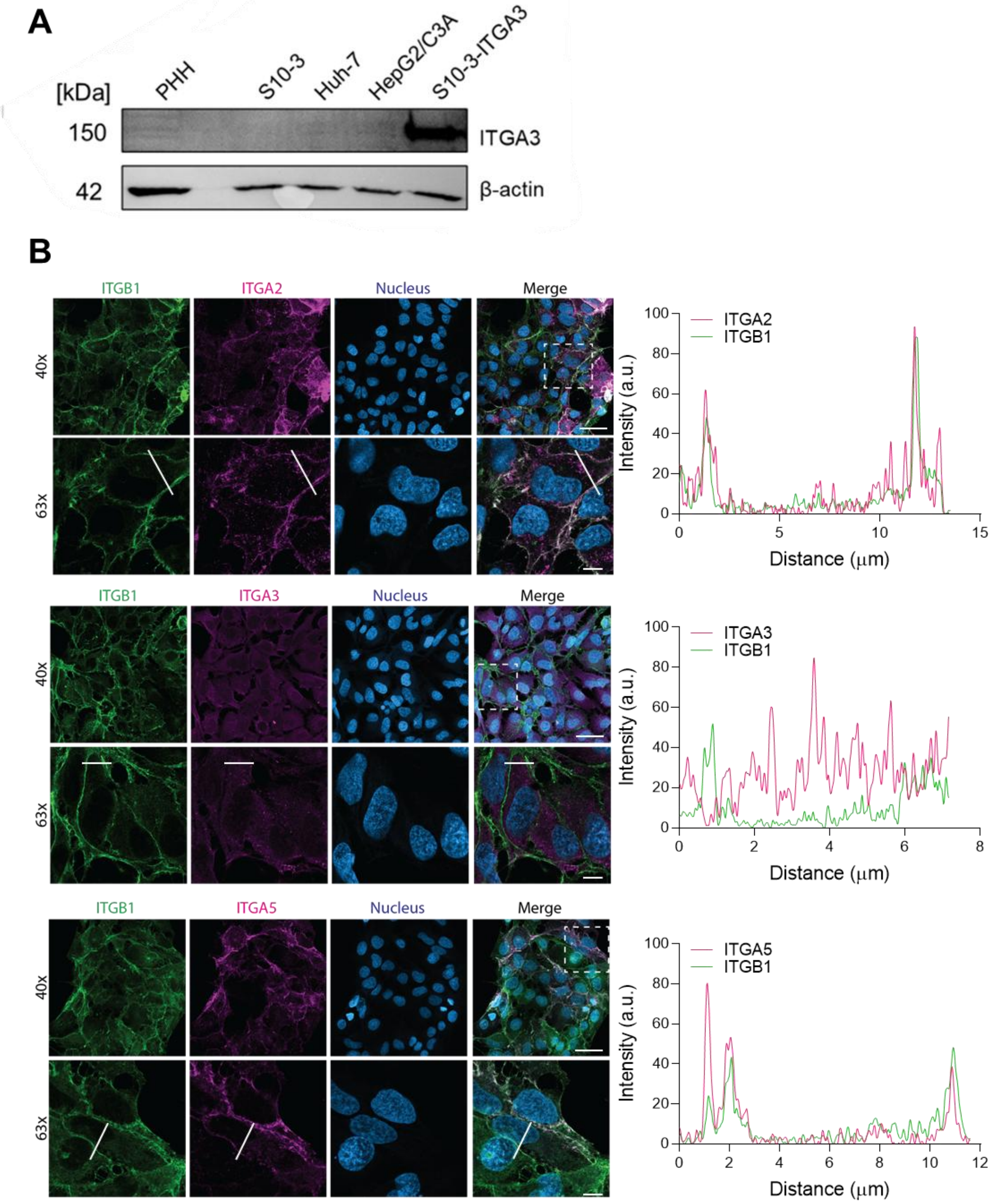

Supplementary Figure 3: Expression and cellular distribution of integrins in hepatoma cells. (A) Western blot analysis of ITGA3 expression in PHH and hepatoma cell lines. Representative image of n = 2. (B) S10-3 WT cells were fixed and co-stained with antibodies against ITGB1 (green) and ITGA2/3/5 (magenta) and counterstained with DAPI (blue). Line graphs on the right show the fluorescence intensities of ITGB1 (green) and ITGA2/3/5 (magenta) measured across the region of interest indicated by the white line in the images shown on the left. The images were taken on a Zeiss Airyscan LSM900 confocal microscope and shown are maximum projections of 2 confocal slices (thickness = 0.25 um). Scale bar = 50 µm at 40x and 10 µm at 63x.

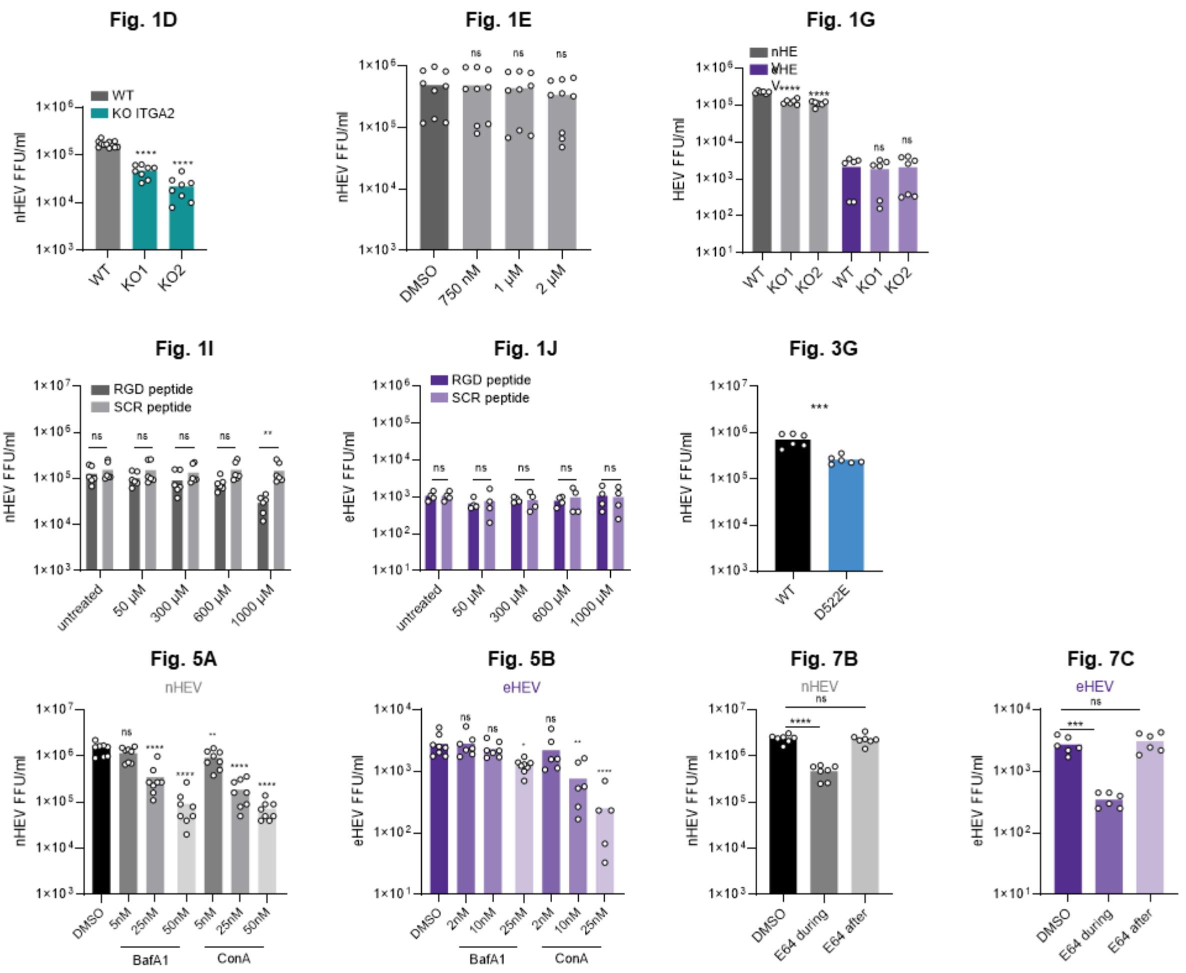

Supplementary Figure 4. Non-normalised data for FFU assays. Non-normalised data shown from the indicated main figures. Statistical analysis was performed as described in the respective main figure legend. **: p <0.01; ***: p<0.001; ****: p<0.0001; ns: non-significant

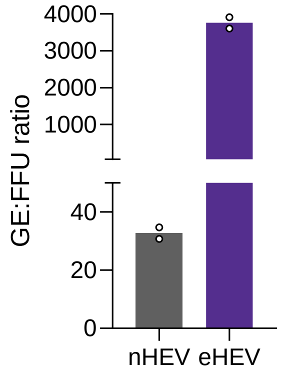

Supplementary Figure 5. Specific infectivity of nHEV and eHEV. Specific infectivity of nHEV and eHEV represented as the ratio of genome equivalents (GE) to FFU. HEV RNA was extracted from gradient-purified virus and quantified by qPCR. HEV infectivity was determined by infecting S10-3 cells and quantifying FFU 5 days post-infection. Images of infected wells were taken with a Zeiss CellDiscoverer 7 microscope and the number of FFU was counted manually. n = 2 replicates from two independent virus productions.

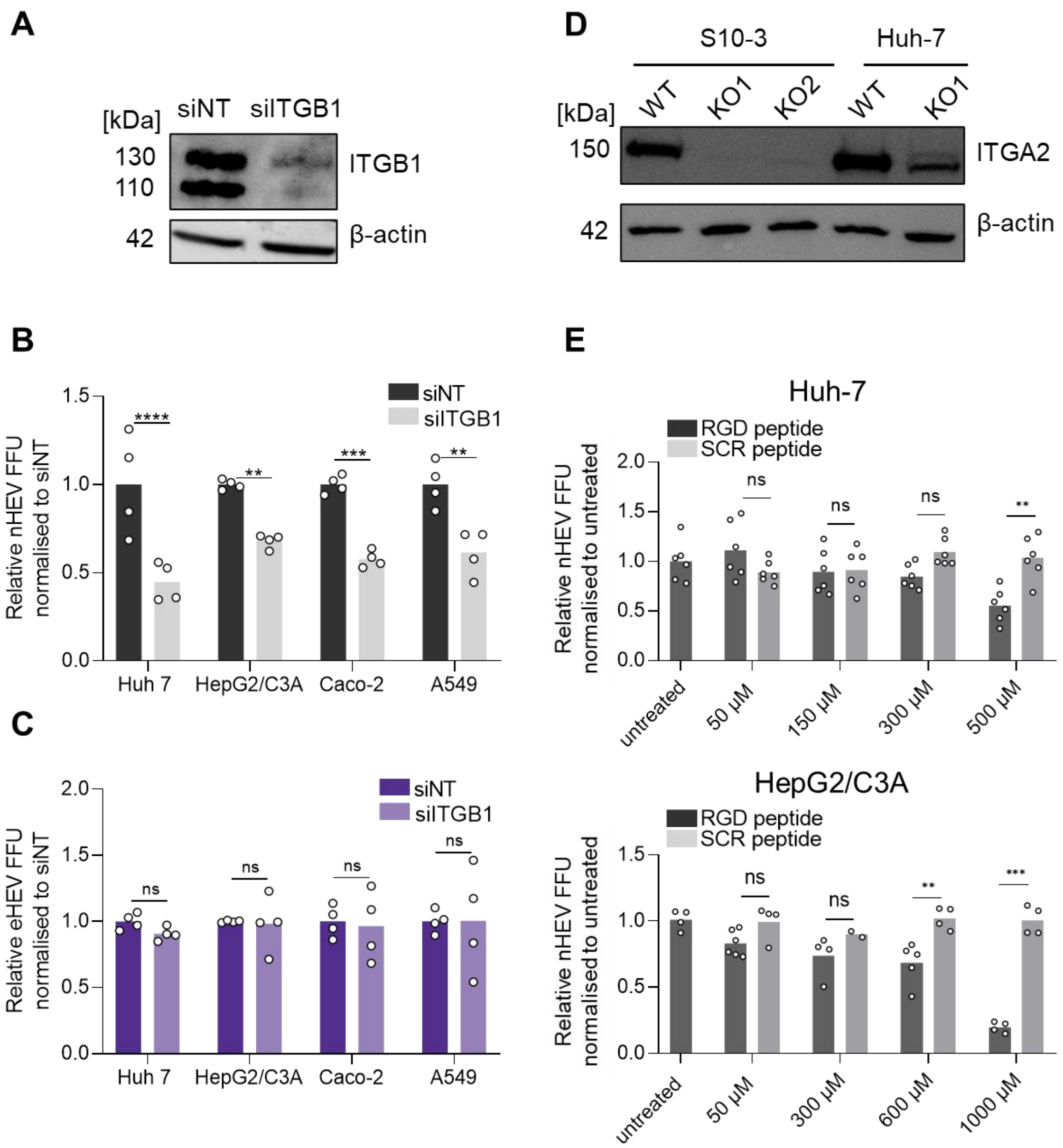

Supplementary Figure 6. Modulation of ITGB1 affects nHEV entry across different cell types. (A) Exemplary Western blot analysis of lysates harvested from HepG2/C3A cells 48 h post-transfection with 100 nM on-target pool siRNAs directed against the ITGB1 gene (siITGB1) or a non-target control (siNT). Representative of n = 2. (B) and (C) siITGB1- and siNT-transfected cells were inoculated with (B) nHEV (MOI = 0.1 GE/cell) or (C) eHEV particles (MOI = 5 GE/cell), 48 h post-transfection. The inoculum was removed after 24 h and HEV infection was quantified by counting ORF2-positive FFUs 5 days post-infection. Images of infected wells were taken with a Zeiss CellDiscoverer 7 microscope and the number of FFU was counted manually. n = 4 replicates from two independent experiments. (D) Western blot analysis of ITGA2 expression in S10-3 and Huh-7 ITGB1 WT and KO cells. Representative of n = 2. (E) Huh-7 and HepG2/C3A cells were infected with nHEV (MOI = 0.1 GE/cell) in the presence of indicated concentrations of an RGD-containing peptide or a scrambled (SCR) control peptide. Infectivity was assessed by staining against the capsid protein ORF2 and counting FFUs 5 days post-infection. n = replicates from two independent experiments. Statistical analysis was performed 2-way ANOVA. **: *p* <0.01; ***: *p*<0.001; ****: p<0.0001; ns: non-significant.

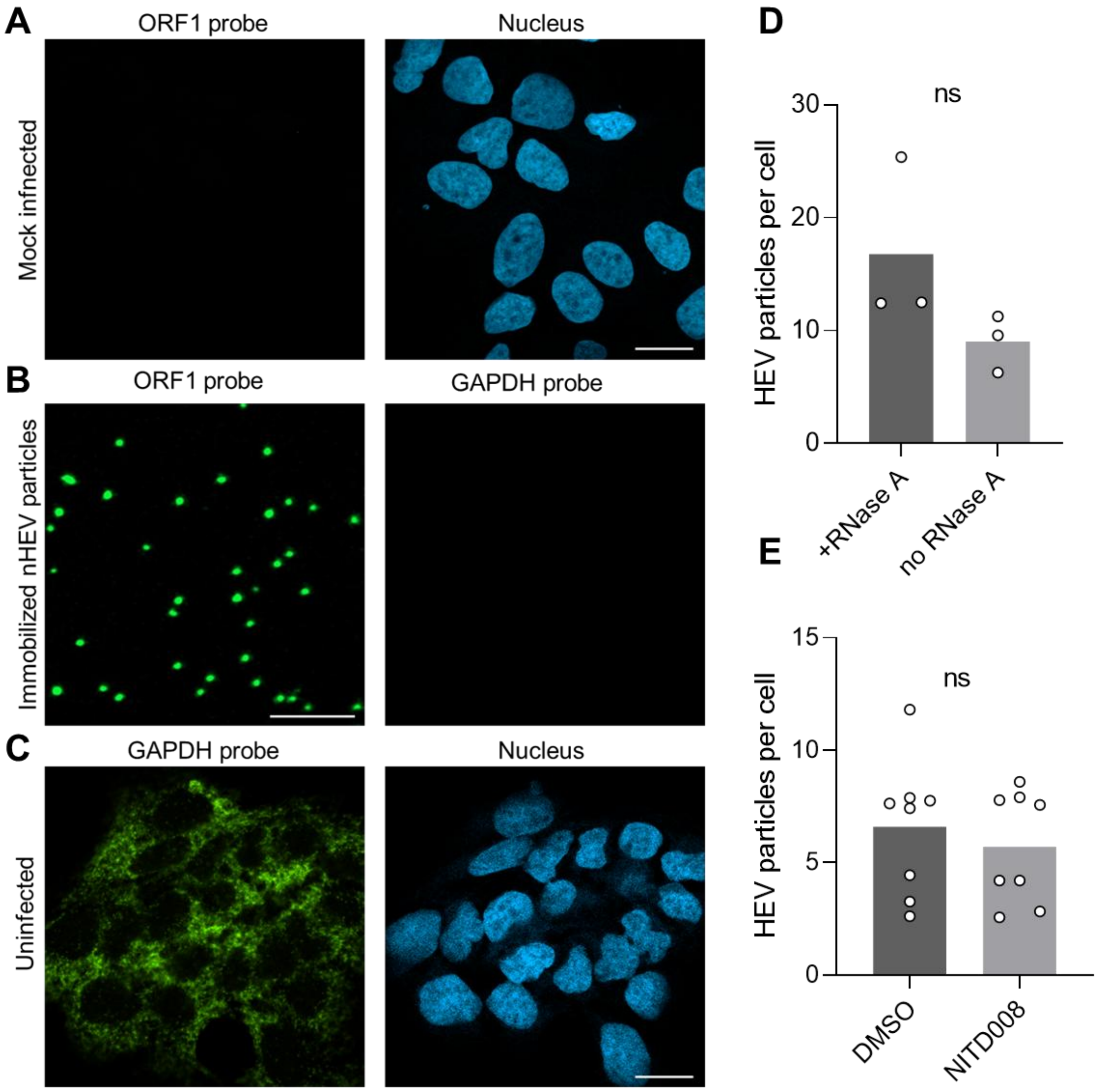

Supplementary Figure 7. Validation of RNAscope as a method for quantifying incoming HEV particles. (A) S10-3 cells were inoculated with PBS for 24 h at 37°C followed by fixation. HEV genomes were detected by RNA-FISH using the ORF1 probe. Scale bar = 20 µm. (B) nHEV particles were immobilized on PEI-coated chamber slides and fixed. After permeabilization, HEV genomes (green) by RNA-FISH using the ORF1 probe. Scale bar = 20 µm. (C) S10-3 cells were seeded on cover slips and fixed after 24 h. GAPDH RNA was detected by RNA-FISH using the GAPDH probe. Scale bar = 20 µm. (A-C) Representative images of n = 2. (D) nHEV particles were pre-incubated with RNase A at 25 µg/ml for 1 h at 37°C. S10-3 cells were then inoculated with treated or untreated virus (MOI = 10 GE/cell) for 6 h. Cells were fixed and HEV genomes were detected by RNA-FISH using the ORF1 probe. The images were taken on a Leica SP8 confocal microscope. HEV particles per cell were calculated by dividing the total number of detected HEV genomes by the number of nuclei in an image frame. n = 3 replicates from one experiment. (E) S10-3 cells were treated with NITD008 or DMSO for 30 min before inoculation with nHEV (MOI = 30 GE/cell). The inoculum was removed 8 h later and replaced with fresh media containing the drug. 24 h later, the cells were fixed and HEV genomes were detected by RNA-FISH using the ORF1 probe. n = 8 replicates from two independent experiments. Images shown in (A) - (C) are single confocal slices taken on a Zeiss Airyscan LSM900 confocal microscope. For (D) and (E), maximum projections of full z-series with a thickness of 10 µm were used for quantification of RNA

and/or capsid particles with CellProfiler. Statistical analysis was performed by Student's t-test. ns, non-significant.

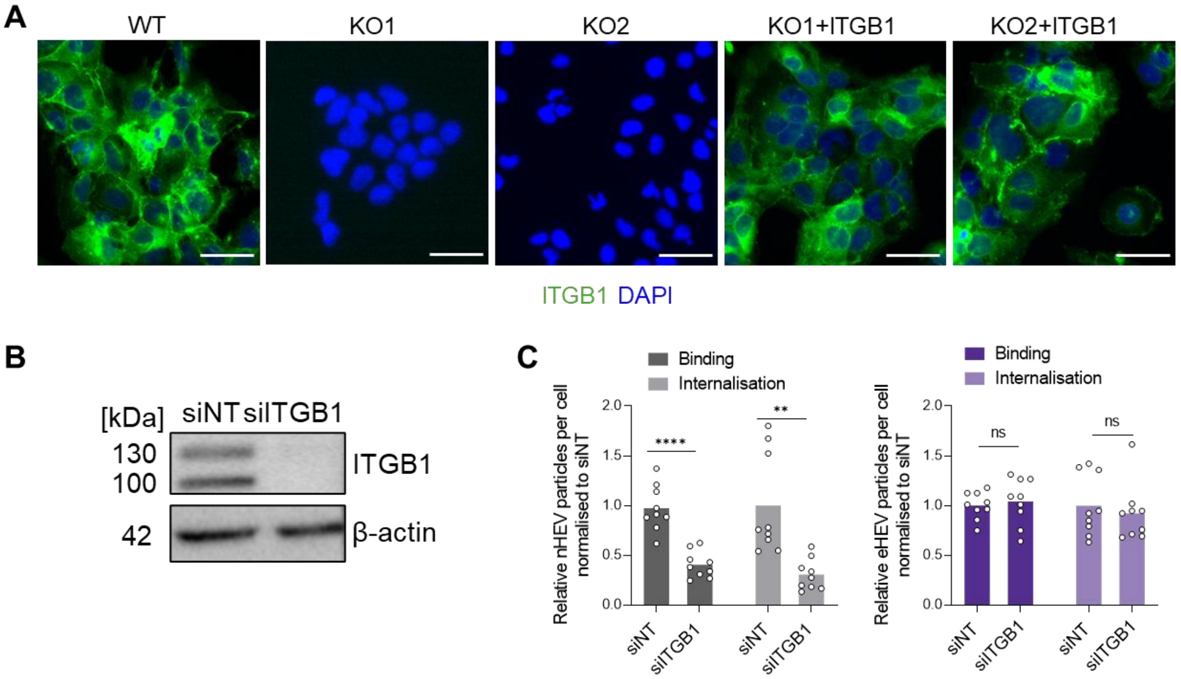

Supplementary Figure 8. Rescue of ITGB1 in KO cells and effect of ITGB1 knockdown in HEV entry in S10-3 cells. (A) ITGB1 expression in ITGB1 KO cells with and without ITGB1 overexpression. S10-3 ITGB1 KO cells were transduced with lentiviruses expressing ITGB1 and selected with puromycin. S10-3 WT and rescued KO cells were fixed and stained with DAPI (blue) and against ITGB1 (green). The representative images (n = 2) were acquired on a Zeiss CellDiscoverer 7 microscope. Scale bar = 50 µm. (B) Western blot analysis of lysates harvested from S10-3 cells 48 h post-transfection with 100 nM on-target pool siRNAs directed against the ITGB1 gene (siITGB1) or a non-target control (siNT). Representative of n = 3. (C) siITGB1- and siNT-transfected S10-3 cells were inoculated with nHEV (left panel) (MOI = 30 GE/cell) or eHEV particles (right panel) (MOI = 20 GE/cell), 48 h post-transfection. The cells were incubated for 2 h and 6 h, respectively, at 4°C to allow particle binding. Cells were then either fixed or the inoculum removed and shifted to incubation at 37°C for 6 h to allow HEV particle internalization. The images were taken on a Zeiss Airyscan LSM900 confocal microscope. Maximum projections of full z-series with a thickness of 10 µm were used for quantification of RNA particles with CellProfiler. n = 9 microscope fields from three independent experiments. Statistical analysis was performed by two-way ANOVA. **: $p < 0.01$; ****: $p < 0.0001$; ns, non-significant.

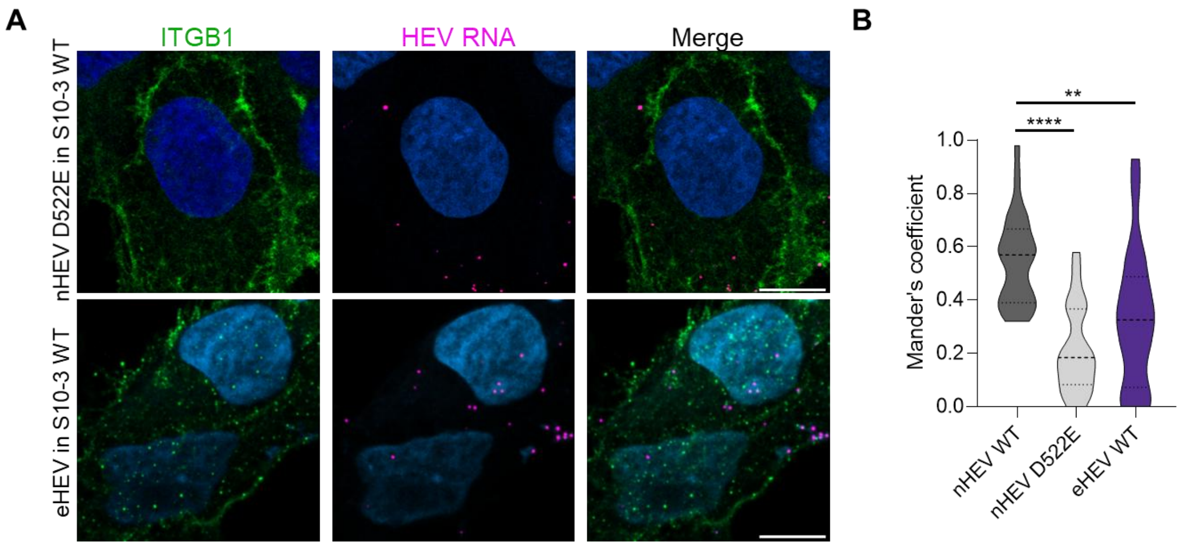

Supplementary Figure 9. Colocalization analysis of nHEV and eHEV particles with ITGB1 upon virus binding. (A) Prechilled S10-3 WT cells were inoculated with nHEV D522E mutant (MOI = 30 GE/cell) or eHEV (MOI = 20 GE/cell) and incubated on ice for 2 h or 6 h, respectively. Following fixation, HEV particles (magenta) were visualised by RNA-FISH followed by IF using an antibody against ITGB1 (green) and counterstain using DAPI (blue). The images were taken on a Zeiss Airyscan LSM900 confocal microscope and shown are maximum projections of single confocal slices. Scale bar = 10 µm. (B) Mander's coefficients of HEV RNA colocalising with ITGB1 were determined using the "Colocalisation" module in Zen 2.1. 20 - 30 cells were analysed for each experimental condition. Statistical analysis was performed by one-way ANOVA. **: *p* <0.01; ****: p<0.0001.

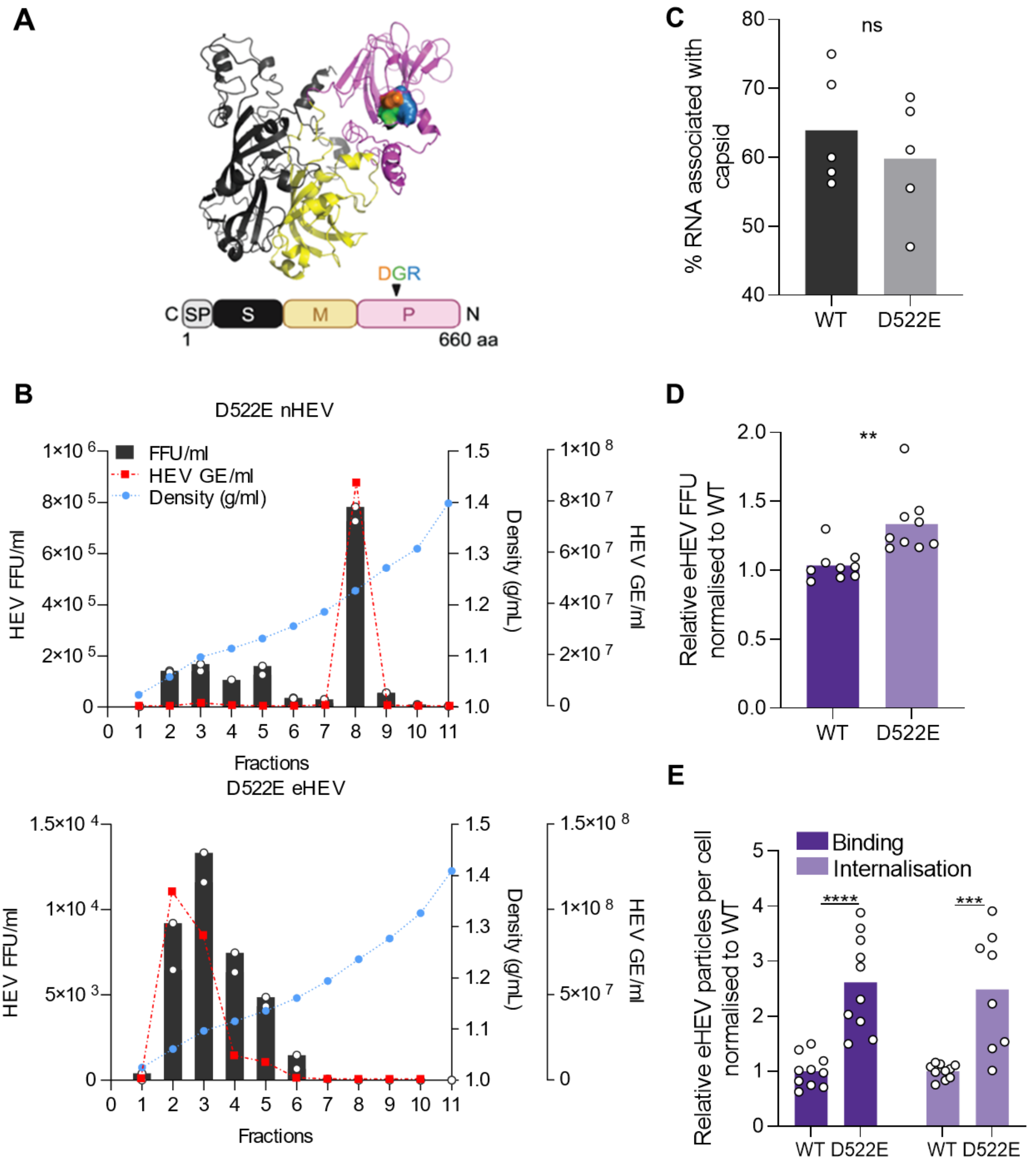

Supplementary Figure 10. Characterization of D522E mutant. (A) Ribbon diagram of ORF2 monomer structure (PDB ID: 2ZTN) with the DGR sequence highlighted by colored spheres. SP = signal peptide, S = shell domain, M = middle domain. P = protrusion domain. Diagram was generated using PYMOL. (B) Buoyant density of D522E HEV virions in cell lysates (upper panel) or released into supernatant fluids (lower panel) of S10-3 cells electroporated with HEV RNA purified through isopycnic iodixanol gradient centrifugation. HEV RNA in fractions was determined by qRT-PCR. HEV infectivity was determined by infecting S10-3 cells and quantifying FFU 5 days post-infection. Images of infected wells were taken with a Zeiss CellDiscoverer 7 microscope and the number of FFU was counted manually. n = 2 replicates from one virus production. (C) Non-enveloped WT and D522E particles were immobilized on PEI-coated chamber slides and fixed with 4% PFA. After permeabilization with 0.1% Triton-X100, capsids were detected by immunofluorescence staining and HEV genomes by RNA-FISH using the ORF1 probe. Shown are the percentages of HEV genomes colocalising with HEV capsids out of total number of detected genomes per cell. n = 5 replicates from two independent virus productions. (D) S10-3 cells were infected with equivalent copies of enveloped WT or D522E mutant virus (MOI = 5 GE/cell). Infectivity was assessed by staining against the capsid protein ORF2 and quantifying FFUs 5 days post-infection. n = 9 replicates from three independent experiments. (E) Prechilled S10-3 cells were inoculated with WT or D522E eHEV particles (MOI = 20 GE/cell) and incubated for 6 h on ice to

allow binding. For internalization, the inoculum was removed after binding and the cells were shifted to 37 °C for 12 h. After binding or internalization, cells were fixed and HEV genomes detected by RNA-FISH using the ORF1 probe. n = 8 microscope fields from three independent experiments. Statistical analysis was performed by Students' t-test (C, D) or two-way ANOVA (E). **: $p<0.01$; ***: $p<0.001$; ****: p<0.0001; ns, non-significant.

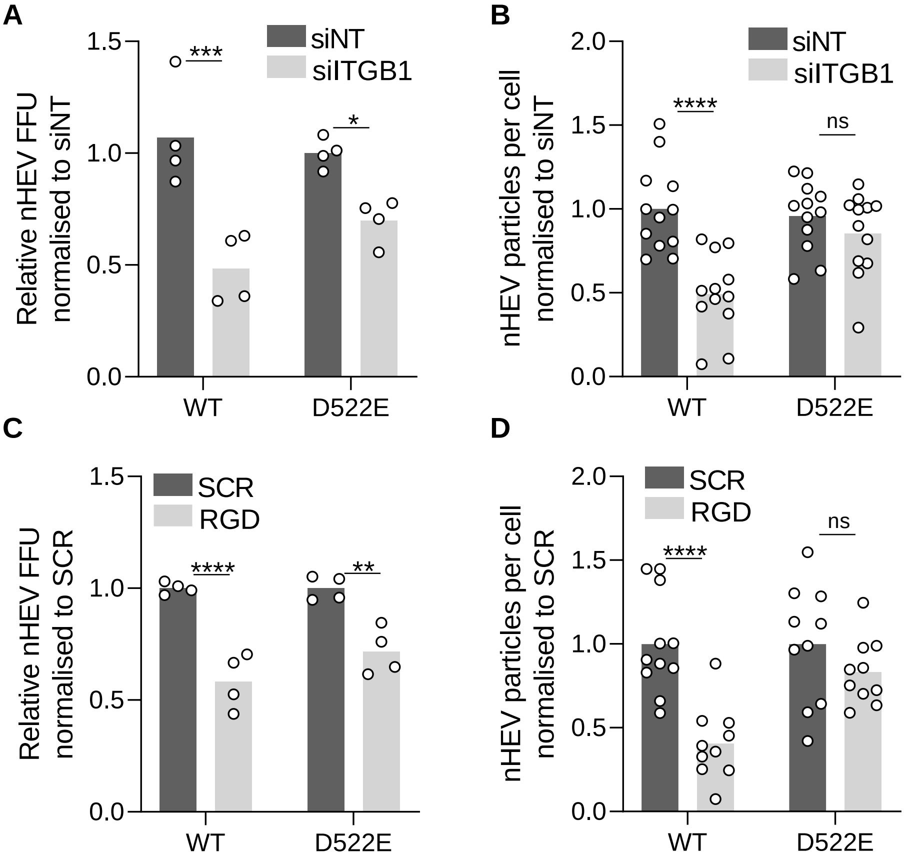

Supplementary Figure 11. The D522E mutation renders nHEV less sensitive to ITGB1 depletion and inhibition. (A) siITGB1- and siNT-transfected S10-3 cells were inoculated with WT or D522E nHEV (MOI = 0.1 GE/cell) 48 h post-transfection. The inoculum was removed after 2 h and HEV infection was quantified by counting ORF2-positive FFUs 5 days post-infection. Images of infected wells were taken with a Zeiss CellDiscoverer 7 microscope and the number of FFU was counted manually. n = 4 replicates from two independent experiments. (B) siITGB1- and siNT-transfected S10-3 cells were inoculated with WT or D522E nHEV (MOI = 30 GE/cell) 48 h post-transfection. The cells were incubated for 2 h at 4°C to allow particle binding. Cells were then fixed and HEV genomes were detected by RNA-FISH using the ORF1 probe. The images were taken on a Zeiss Airyscan LSM900 confocal microscope. The detected HEV genomes were quantified using CellProfiler. n = 12 microscope fields from two independent experiments. (C) S10-3 cells were infected with WT or D522E nHEV (MOI = 0.1 GE/cell) in the presence of 600 µM of an RGD-containing peptide or a scrambled (SCR) control peptide during entry for 6 h. Infectivity was assessed by staining against the capsid protein ORF2 and quantifying FFUs 5 days post-infection. n = 4 replicates from two independent experiments. (D) S10-3 cells were infected with WT or D522E nHEV (MOI = 30 GE/cell) in the presence of 600 µM of an RGD-containing peptide or a scrambled (SCR) control peptide. The cells were incubated for 2 h at 4°C to allow particle binding. Cells were then fixed and HEV genomes were detected by RNA-FISH using the ORF1 probe. The images were taken on a Zeiss Airyscan LSM900 confocal microscope. The detected HEV genomes were quantified using CellProfiler. n = 10 microscope fields from two independent experiments. Statistical analysis was performed by two-way ANOVA. *: p <0.05; ***: $p<0.001$; ****: p<0.0001; ns, non-significant.

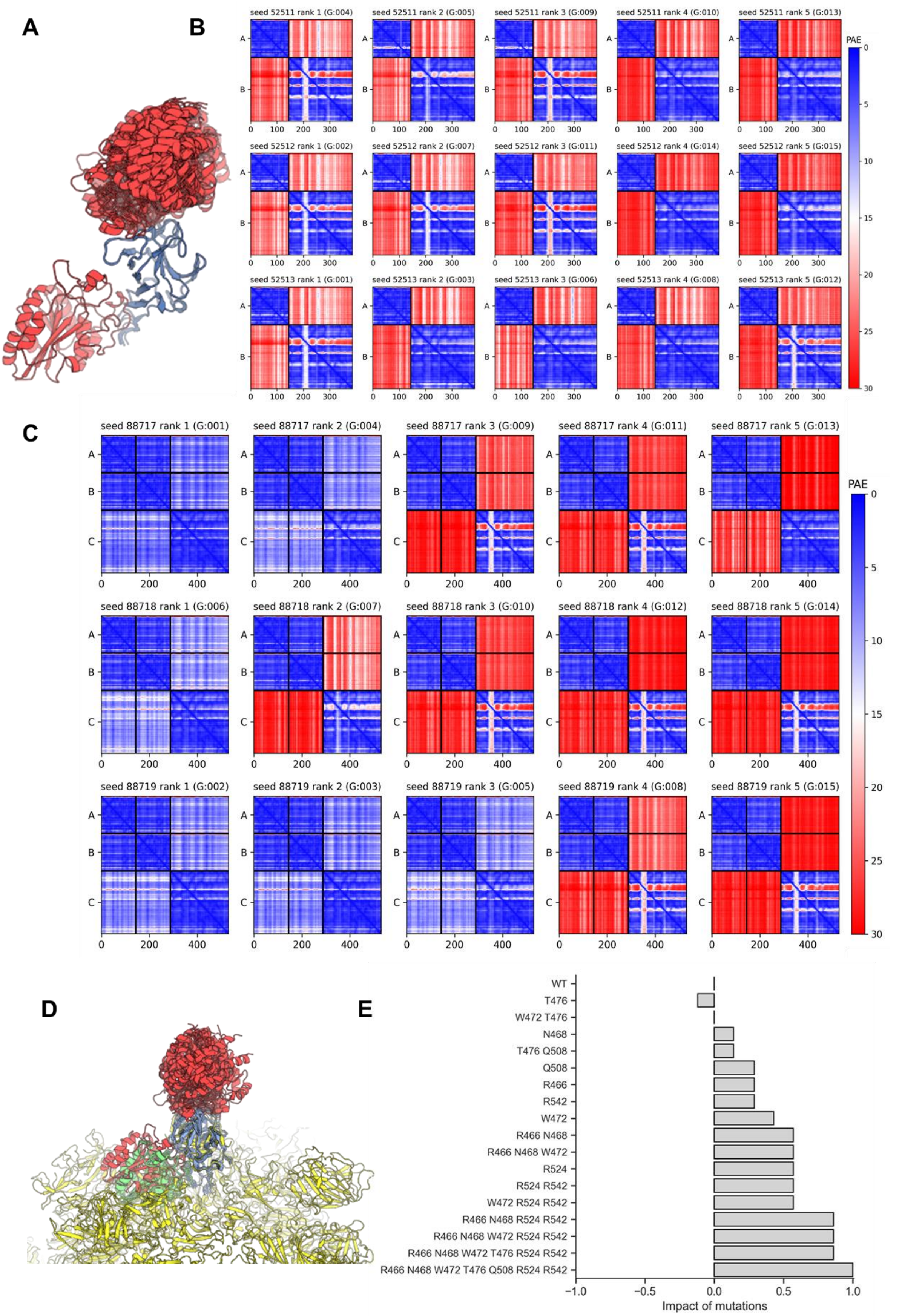

Supplementary Figure 12. Comprehensive modelling of protein interaction between ITGB1$_{139\text{-}380}$ and ORF2 P domain. (A) Spatial arrangement of ITGB1$_{139\text{-}380}$ models (red) relative to a monomer of

the ORF2 P domain (dark blue). (B) PAE matrices for each of the 15 models, demonstrating no low PAE values between ORF2-P (chain A) and $ITGB1_{139-380}$ (chain B). (C) PAE matrices for each model, where A and B correspond to ORF2-P monomers and C represents $ITGB1_{139-380}$ showing six matrices with low PAE values between the dimer of ORF2-P and $ITGB1_{139-380}$. (D) Superposition of ORF2-P dimer models (dark blue) with $ITGB1_{139-380}$ (green: favorable docking, red: unfavorable) and the HEV virus-like particle structure (6LAT). This superposition suggests that well-docked $ITGB1_{139-380}$ models occupy a region within the capsid that would be inaccessible without conformational changes enabled by the flexible M-P domain linker. (E) Impact of alanine mutations on model quality, quantified by the number of PAE matrices exhibiting low PAEinter values (1: complete degradation of binding prediction, -1: complete improvement in binding prediction).

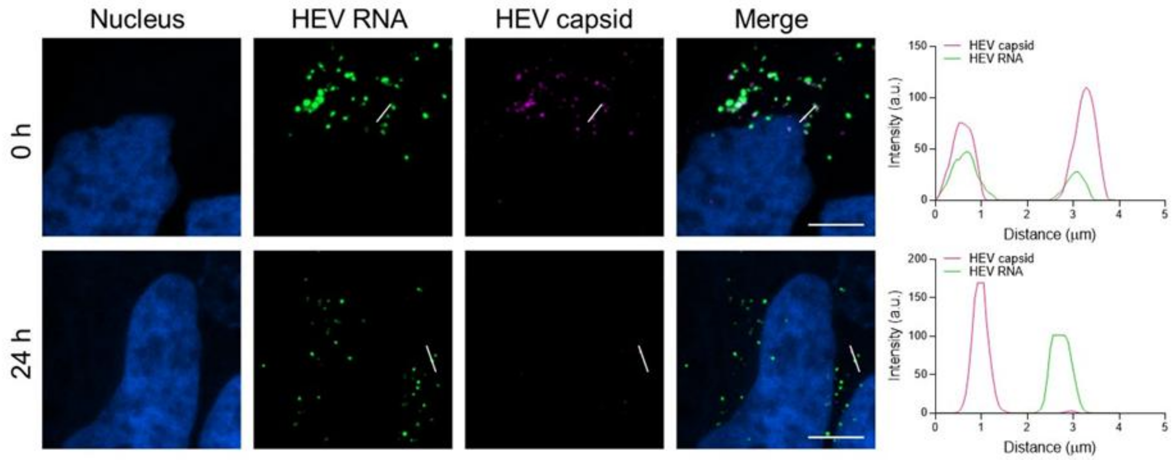

Supplementary Figure 13. Co-detection of eHEV capsid and genome. S10-3 cells were inoculated with eHEV (MOI = 20 GE/cell) and incubated for 6 h at 4 °C to allow binding (upper image row) followed by inoculum removal and 24 h at 37°C for internalisation (lower image row). After fixation, cells were stained for HEV capsids (magenta) and HEV genomes (green) detected by RNA-FISH using the ORF1 probe. Line graphs on the right show the fluorescence intensities of capsids and genomes measured across the region of interest indicated by the white line in the images shown on the left. Images are representatives of n = 6. Scale bar = 5 µm.

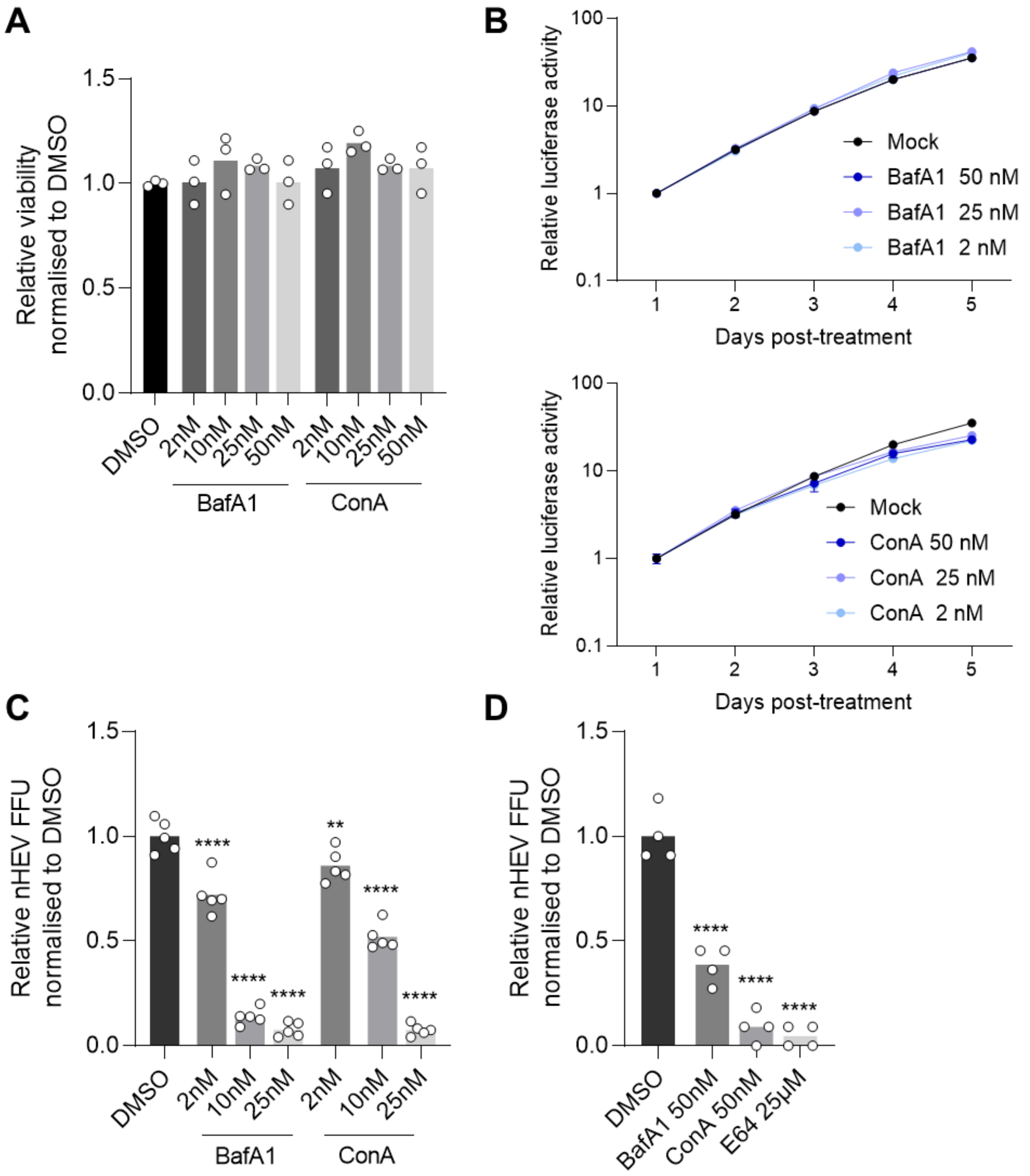

Supplemental Figure 14. Effect of endocytic inhibitors on cell viability, viral replication and HEV infection in HepG2/C3A and PHH. (A) S10-3 cells were treated with indicated concentrations of bafilomyin A (BafA1), concanamyin A (ConA) or DMSO for 18 h and cell viability was measured by an MTT assay. n = 3 replicates from one experiment. (B) S10-3 cells were electroporated with an HEV-GLuc subgenomic replicon and treated with the inhibitors in (A). HEV replication was quantified by measuring luciferase activity in the supernatant during days 1 to 4 post-electroporation. n = replicates from two independent experiments. (C) HepG2/C3A cells were treated with indicated concentrations of BafA1, ConA or DMSO (mock) for 30 min prior to infecting with nHEV (MOI = 0.1 GE/cell). Drugs and virus were removed after 24 h and HEV infection was quantified by counting ORF2-positive FFUs 5 days post-infection. Images of entire infected wells were taken with a Zeiss CellDiscoverer 7 microscope and the number of FFU was counted manually. n = 4 replicates from two independent experiments. (D) Primary human hepatocytes (PHH) were treated with indicated concentrations of BafA1, ConA and E64 in the same manner as in (C) and inoculated with nHEV (MOI = 0.5 GE/cell). FFUs were quantified 3 days post-infection. n = 5 replicates from one PHH doner. Images of entire infected wells were taken with a Zeiss CellDiscoverer 7 microscope and the number of FFU was counted manually. Statistical analysis was performed by one-way ANOVA. **: p <0.01; ****: p<0.0001.

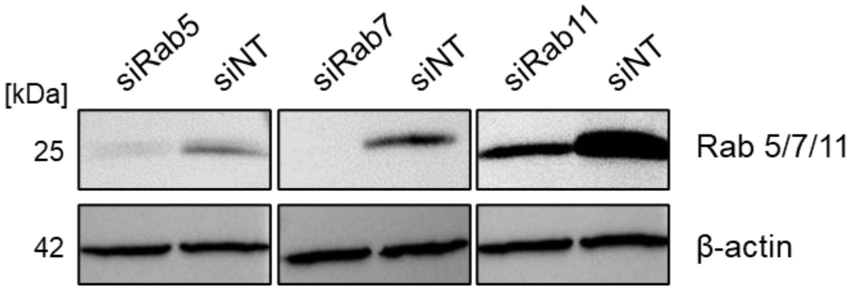

Supplemental Figure 15. siRNA-mediated knockdown of Rab5, Rab7 and Rab11. Western blot analysis of lysates harvested from S10-3 cells 48 h post-transfection with 100 nM on-target pool siRNAs directed against Rab5, Rab7, Rab11, or a non-target control (siNT). Representative of n = 2.

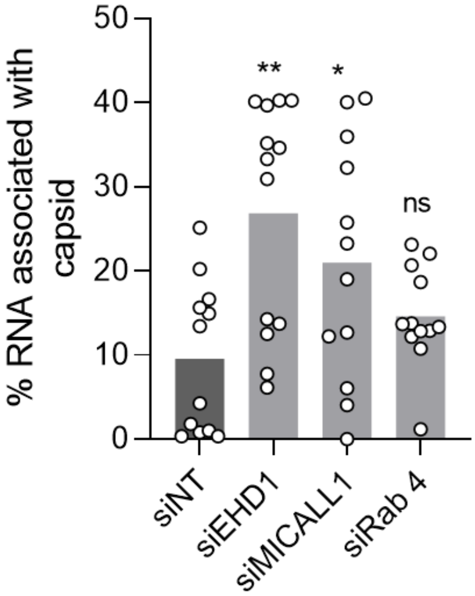

Supplementary Figure 16. Knockdown of components of the recycling endosome impairs nHEV uncoating. siRNA-transfected S10-3 cells were inoculated with nHEV (MOI = 30 GE/cell) and incubated for 8 h at 37 °C 48 h post-transfection. 24 h post-inoculation, HEV capsid and HEV genomes were detected by immunofluorescence staining against the capsid protein and RNA-FISH using the ORF1 probe, respectively. n = 12 microscope fields from two independent experiments Statistical analysis was performed by one-way ANOVA. *: $p < 0.05$; **: $p < 0.01$; ns, non-significant.

Supplementary References

1 Shukla, P. *et al.* Adaptation of a genotype 3 hepatitis E virus to efficient growth in cell culture depends on an inserted human gene segment acquired by recombination. *J Virol* **86**, 5697-5707, doi:10.1128/JVI.00146-12 (2012).
