# Supplementary material for: Integrin beta 1 facilitates non-enveloped hepatitis E virus cell entry through the recycling endosome
Source: Nat Commun. 2025 Jun 26;16:5403. doi: 10.1038/s41467-025-61071-y (PMC12202797; doi:10.1038/s41467-025-61071-y)
Supplement: Supplementary file 2 — Reporting summary [file 41467_2025_61071_MOESM2_ESM.pdf]

nature portfolio

Corresponding author(s): Viet Loan Dao Thi

Last updated by author(s): 2025.05.30

Reporting Summary

Nature Portfolio wishes to improve the reproducibility of the work that we publish. This form provides structure for consistency and transparency in reporting. For further information on Nature Portfolio policies, see our Editorial Policies and the Editorial Policy Checklist.

Statistics

For all statistical analyses, confirm that the following items are present in the figure legend, table legend, main text, or Methods section.

n/a	Confirmed	
☐	☒	The exact sample size (*n*) for each experimental group/condition, given as a discrete number and unit of measurement
☐	☒	A statement on whether measurements were taken from distinct samples or whether the same sample was measured repeatedly
☐	☒	The statistical test(s) used AND whether they are one- or two-sided<br>*Only common tests should be described solely by name; describe more complex techniques in the Methods section.*
☐	☒	A description of all covariates tested
☐	☐	A description of any assumptions or corrections, such as tests of normality and adjustment for multiple comparisons
☐	☒	A full description of the statistical parameters including central tendency (e.g. means) or other basic estimates (e.g. regression coefficient) AND variation (e.g. standard deviation) or associated estimates of uncertainty (e.g. confidence intervals)
☐	☒	For null hypothesis testing, the test statistic (e.g. *F*, *t*, *r*) with confidence intervals, effect sizes, degrees of freedom and *P* value noted<br>*Give P values as exact values whenever suitable.*
☒	☐	For Bayesian analysis, information on the choice of priors and Markov chain Monte Carlo settings
☒	☐	For hierarchical and complex designs, identification of the appropriate level for tests and full reporting of outcomes
☒	☐	Estimates of effect sizes (e.g. Cohen's *d*, Pearson's *r*), indicating how they were calculated

Our web collection on statistics for biologists contains articles on many of the points above.

Software and code

Policy information about availability of computer code

Data collection	The LC–MS/MS data were acquired by coupling an Ultimate 3000 HPLC to an Orbitrap Exploris 480 mass spectrometer. The mass spectrometer was operated in in data-independent mode (DIA).<br>ZEISS ZEN and Leica SP8 was used to acquire confocal microscopy images. Western blots were imaged using the ChemoStar Touch ECL & Fluorescence Imager software. CellDiscoverer 7 was used to acquire brightfield images for quantification of FFUs.
Data analysis	All DIA raw data files were analysed with a direct DIA workflow using Spectronaut 17.6 (Biognosys, Zurich, Switzerland). The Uniprot Homo sapiens reference proteome database was used for the Pulsar search. ZEISS ZEN desk was used to process confocal microscopy images. Processed images were analyzed using CellProfiler 4.1.3 or ImageJ version 1.54k. Graphpad PRISM version 8.0 was used for statistical analysis.

For manuscripts utilizing custom algorithms or software that are central to the research but not yet described in published literature, software must be made available to editors and reviewers. We strongly encourage code deposition in a community repository (e.g. GitHub). See the Nature Portfolio guidelines for submitting code & software for further information.

Data

Policy information about availability of data

All manuscripts must include a data availability statement. This statement should provide the following information, where applicable:

- Accession codes, unique identifiers, or web links for publicly available datasets
- A description of any restrictions on data availability
- For clinical datasets or third party data, please ensure that the statement adheres to our policy

Proteomic data sets have been deposited to PRIDE with project accession: PXD052479. Access link: https://www.ebi.ac.uk/pride/review-dataset/be8b4508e9ba4790ad1894c4b79ec52f. Token: 7liS3O3cZFvn

Research involving human participants, their data, or biological material

Policy information about studies with human participants or human data. See also policy information about sex, gender (identity/presentation), and sexual orientation and race, ethnicity and racism.

Reporting on sex and gender	No human participants were involved in this study.
Reporting on race, ethnicity, or other socially relevant groupings	No human participants were involved in this study.
Population characteristics	No human participants were involved in this study.
Recruitment	No human participants were involved in this study.
Ethics oversight	No human participants were involved in this study.

Note that full information on the approval of the study protocol must also be provided in the manuscript.

Field-specific reporting

Please select the one below that is the best fit for your research. If you are not sure, read the appropriate sections before making your selection.

☒ Life sciences ☐ Behavioural & social sciences ☐ Ecological, evolutionary & environmental sciences

For a reference copy of the document with all sections, see nature.com/documents/nr-reporting-summary-flat.pdf

Life sciences study design

All studies must disclose on these points even when the disclosure is negative.

Sample size	No sample-size calculations were performed.
Data exclusions	No data were excluded from the analyses.
Replication	Experiments revealing differences in measured data were repeated at least once and all results were successfully replicated.
Randomization	The study includes no experiments dependent on allocation of samples/organisms/participants into experimental groups.
Blinding	The study includes no experiments dependent on group allocation and blinding is thus not relevant.

Reporting for specific materials, systems and methods

We require information from authors about some types of materials, experimental systems and methods used in many studies. Here, indicate whether each material, system or method listed is relevant to your study. If you are not sure if a list item applies to your research, read the appropriate section before selecting a response.

Materials & experimental systems

n/a	Involved in the study
☐	☒ Antibodies
☐	☒ Eukaryotic cell lines
☒	☐ Palaeontology and archaeology
☒	☐ Animals and other organisms
☒	☐ Clinical data
☒	☐ Dual use research of concern
☒	☐ Plants

Methods

n/a	Involved in the study
☒	☐ ChIP-seq
☒	☐ Flow cytometry
☒	☐ MRI-based neuroimaging

Antibodies

Antibodies used

α-ITGB1 (Santa Cruz, #sc-53711); α-ITGA2 (Abcam, ab181549); α-ITGA3 (Millipore, AB1920); α-ITGA5 1:500 (Abcam, EPR7854), α-ITGA6 1:500 (St.John's Laboratory, STJA0014641); α-ITGA1 (Abcam, ab200570); α-actin (Sigma, A2228) ; α-Rab 7 (Abcam, EPR7589) ; α-Rab 11 (Abcam, RRID AB_2533987); α-Rab 5 (antibodies-online, ABIN361846) , α-tubulin (Sigma, T9026); α-FAK (Santa Cruz, sc-271126); α-Na/K+ ATPase (Millipore, 05-369); α-HEV ORF2 (clone 1E6, Millipore, MAB8002), α-HEV ORF2 (pAB, kind gift from Rainer Ulrich, FLI), secondary antibodies conjugated to Alexa Flour (Thermo Fisher), secondary antibodies conjugated to HRP (Jackson ImmunoResearch)

Validation

All antibodies have been validated by the manufacturers and previous experiments.

Eukaryotic cell lines

Policy information about cell lines and Sex and Gender in Research

Cell line source(s)

Primary human hepatocytes were purchased from PRIMACYST (Lot #: CHM2225-HE-Z).
S10-3 cells were a kind gift from Suzanne Emerson, NIH.
HepG2, Huh-1, Huh-6, Hep3b, PLC/PRE/5, HLF, HLE, Snu172, Snu387 cells were kind gifts from Marco Binder, DKFZ.
HepG2/C3A were purchased from ATCC and Huh-7 cells (Nakabayashi et al., Cancer Res) were a kind gift from Darius Moradpour.
A549 cells where a kind gift from Britta Brüger, Heidelberg University
Caco-2 cells were a kind gift from Volker Lohmann, Heidelberg University

Authentication

Non-purchased cell lines used were authenticated by Multiplexion GmbH

Mycoplasma contamination

All cells tested negative for mycoplasma contamination by either Eurofins Genomics or Multiplexion GmbH

Commonly misidentified lines
(See ICLAC register)

Name any commonly misidentified cell lines used in the study and provide a rationale for their use.

Plants

Seed stocks

Report on the source of all seed stocks or other plant material used. If applicable, state the seed stock centre and catalogue number. If plant specimens were collected from the field, describe the collection location, date and sampling procedures.

Novel plant genotypes

Describe the methods by which all novel plant genotypes were produced. This includes those generated by transgenic approaches, gene editing, chemical/radiation-based mutagenesis and hybridization. For transgenic lines, describe the transformation method, the number of independent lines analyzed and the generation upon which experiments were performed. For gene-edited lines, describe the editor used, the endogenous sequence targeted for editing, the targeting guide RNA sequence (if applicable) and how the editor was applied.

Authentication

Describe any authentication procedures for each seed stock used or novel genotype generated. Describe any experiments used to assess the effect of a mutation and, where applicable, how potential secondary effects (e.g. second site T-DNA insertions, mosiacism, off-target gene editing) were examined.
